# Loss of guidewire and its sequelae after central venous catheterization: A case report

**DOI:** 10.1097/MD.0000000000016513

**Published:** 2019-07-19

**Authors:** Shenyu Zhao, Zhe Wang, Yu Zhao

**Affiliations:** Department of Vascular Surgery, First Affiliated Hospital of Chongqing Medical University, Chongqing, China.

**Keywords:** aortic arch, catheters, complications, imaging CT

## Abstract

**Rationale:**

Central venous catheterization is a common tool used to monitor central venous pressure and administer fluid medications in patients undergoing surgery. The loss of a broken guide wire into the circulation is a rare and preventable complication. Here, we report a peculiar case of a missed guidewire puncturing the aortic arch and cerebrum.

**Patient concerns:**

A 53-year-old man with complaints of an intermittent headache and right swollen ankle following central venous catheterization.

**Diagnoses:**

Using computed tomography; the patient was diagnosed with the loss of a guide wire in his body. The guide wire had migrated to the brain and punctured the vascular wall of the aortic arch.

**Interventions:**

Due to the risks of surgery, the patient was advised to have a follow-up visit once every 3 months.

**Outcomes:**

At present, the patient could live like a normal person, although he suffers from intermittent headaches.

**Lessons:**

The loss of a guide wire is a completely preventable complication, provided that a hold on the tip of the wire is maintained during placement, and the correct safety measurements and protocols are followed.

## 1 Introduction

Central venous catheterization (CVC) is a routine technique used in the emergency department and intensive care unit. Central venous catheterizations are necessary for critical patients in special conditions and as a route for parenteral nutrition. Different veins, such as the jugular, subclavian, femoral, and brachial veins, can be chosen depending on the catheter type. The complication rate of CVCs is estimated to be approximately 12% to 15%.^[1,2]^ One of these extremely rare complications is the intravascular loss of a guide wire, which is often found incidentally following a routine X-ray or computed tomography (CT) performed several months after the procedure.^[1,2,3]^ Hereby, we report the migration of a guide wire into the circulation from right femoral vein catheterization and caused damage arch of aorta and cerebrum.

## 2 Case report

A 53-year-old man presented to the outpatient department with complaints of an intermittent headache and a right swollen ankle, which became worse after activity. His history included a craniocerebral trauma related to a fall during alcohol intoxication. In the first quarter of 2015, during the removal of cranial epidural hemorrhage, percutaneous catheterization of central veins via the right femoral vein was attempted because of the need for intravenous infusion. After the surgery, the patient was sent to the intensive care unit for postoperative care. A few days later, the patient was discharged home; he demonstrated a slight weak muscular tension in his right upper extremity and had difficulty in speaking extended sentences. Approximately 1 and a half months after the surgery, the patient was re-examined by CT. The CT scan showed that 1 guidewire had ascended in the superior vena cava and the heart. Despite this finding, the hospital was unable to perform the surgery to remove the guide wire, and the doctors suggested that he should have a follow-up visit in every few months. The patient ignored the guide wire, took no medication, and continued to lead a normal life. In January 2016, the patient received another CT scan which revealed that the guide wire had arrived at the cerebrum. However, the patient did not have any symptoms of discomfort. The doctors suggested that the patient should go to a better hospital for further treatment.

**Figure 1:**
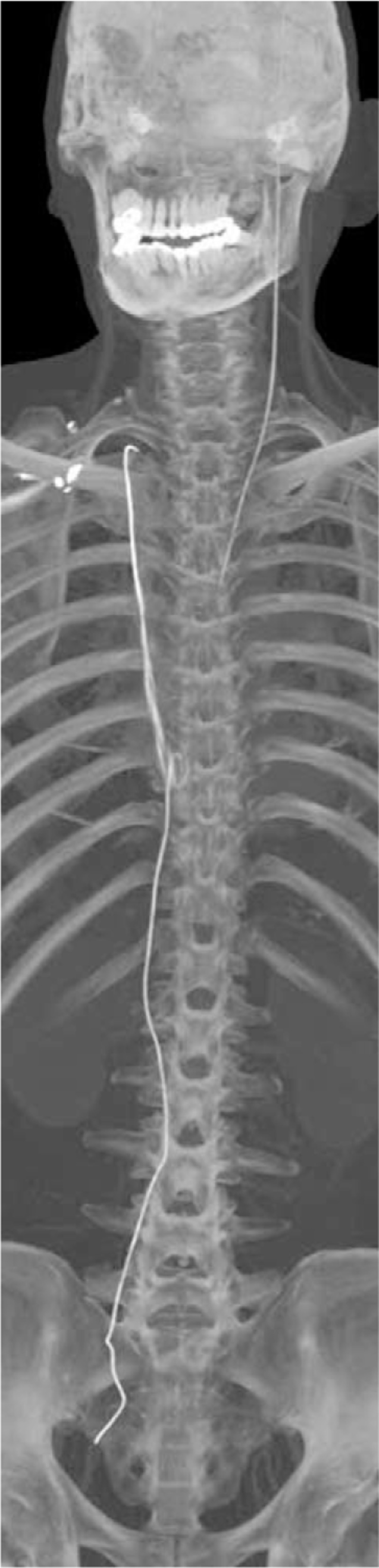
3D reconstruction of computed tomography showing one of guide wire had entered in the right brachiocephalic vena cava and the other one had entered in the cerebrum via the aortic arch.

**Figure 2:**
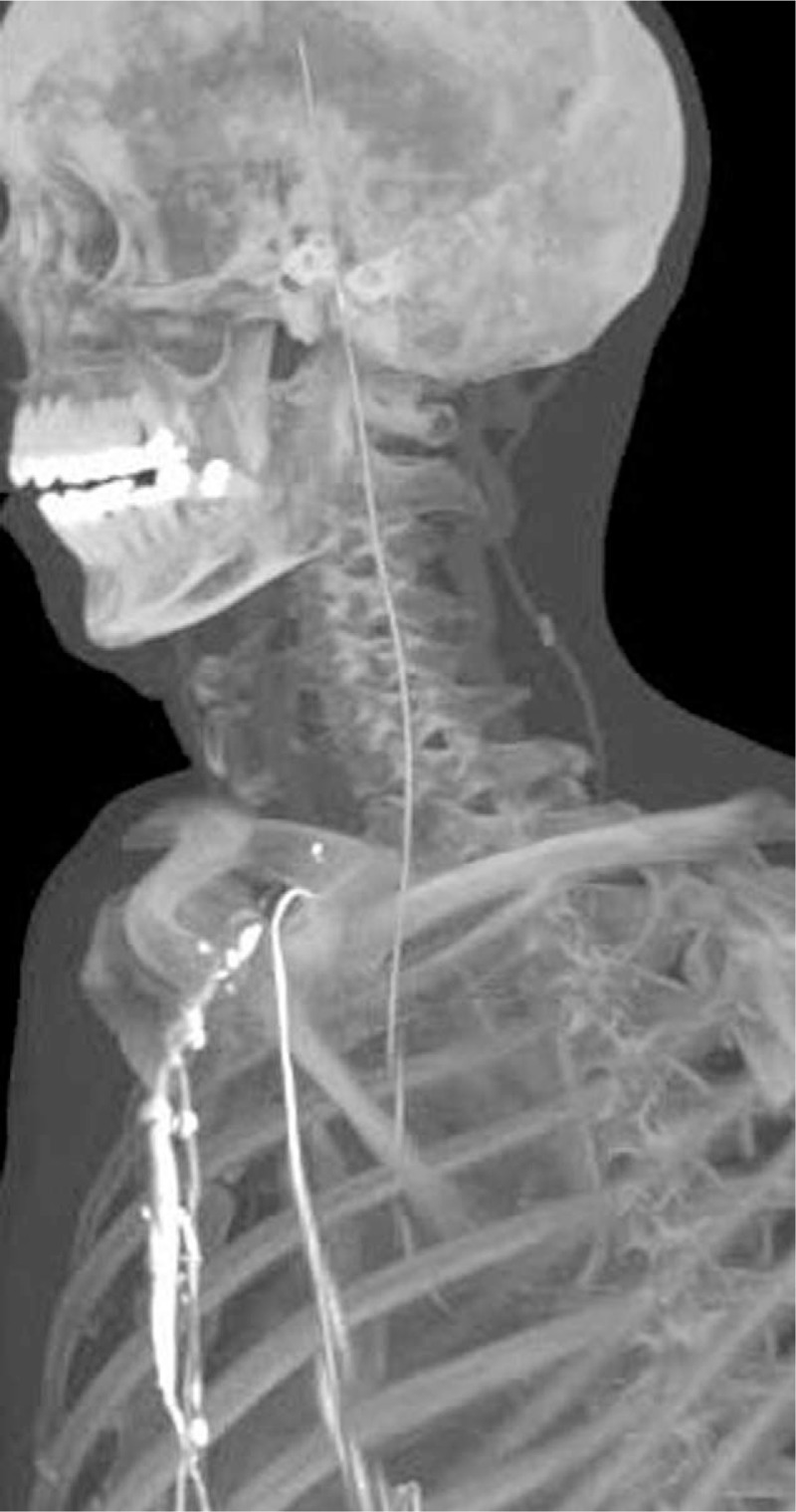
3D reconstruction of computed tomography showing the position of the guide wire in the cerebrum.

**Figure 3:**
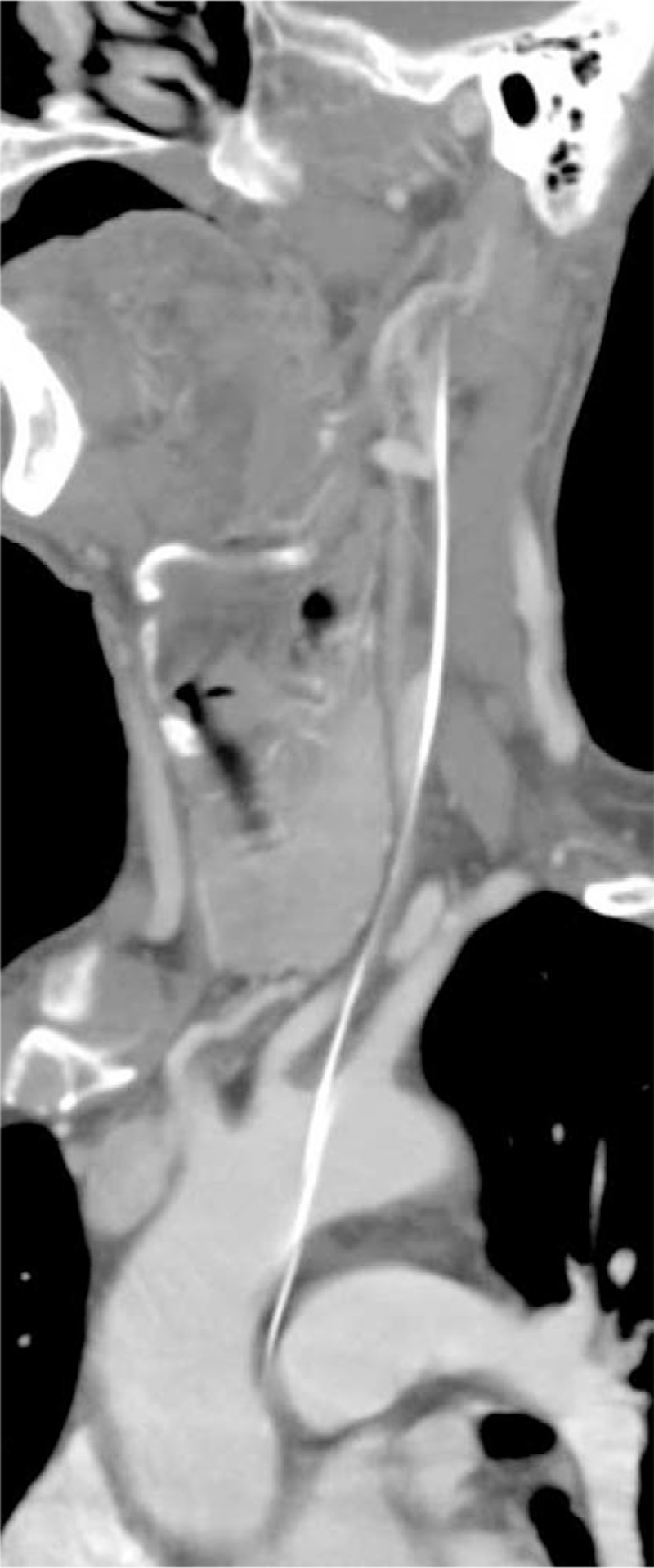
Computed tomography showing guide wire had punctured the vascular wall of the aortic arch to reach the cerebrum.

**Figure 4:**
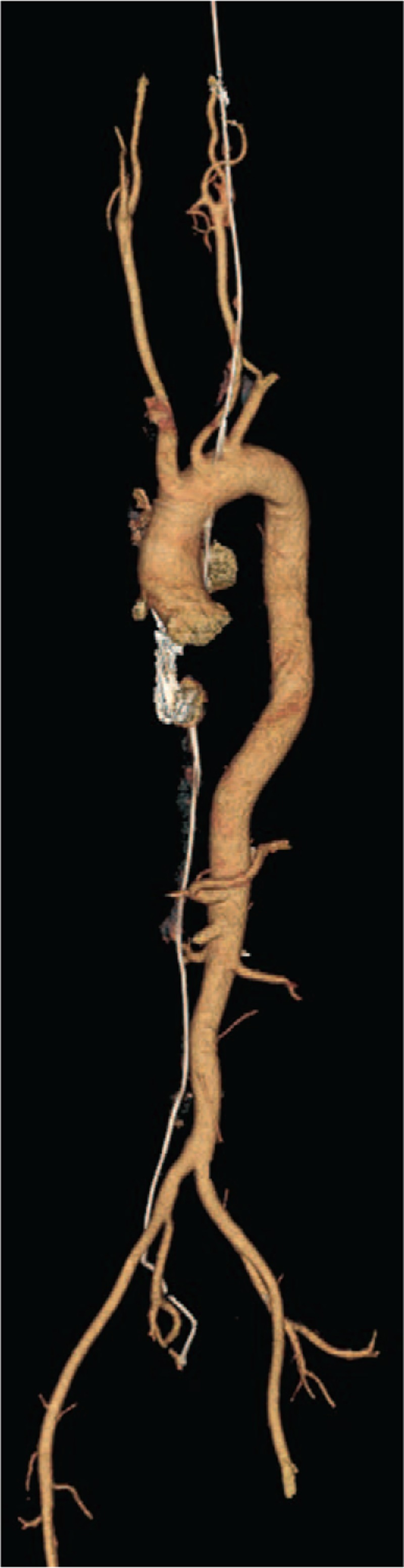
Computed tomography angiography showing guide wire had punctured the vascular wall of the aortic arch to reach the cerebrum.

**Figure 5:**
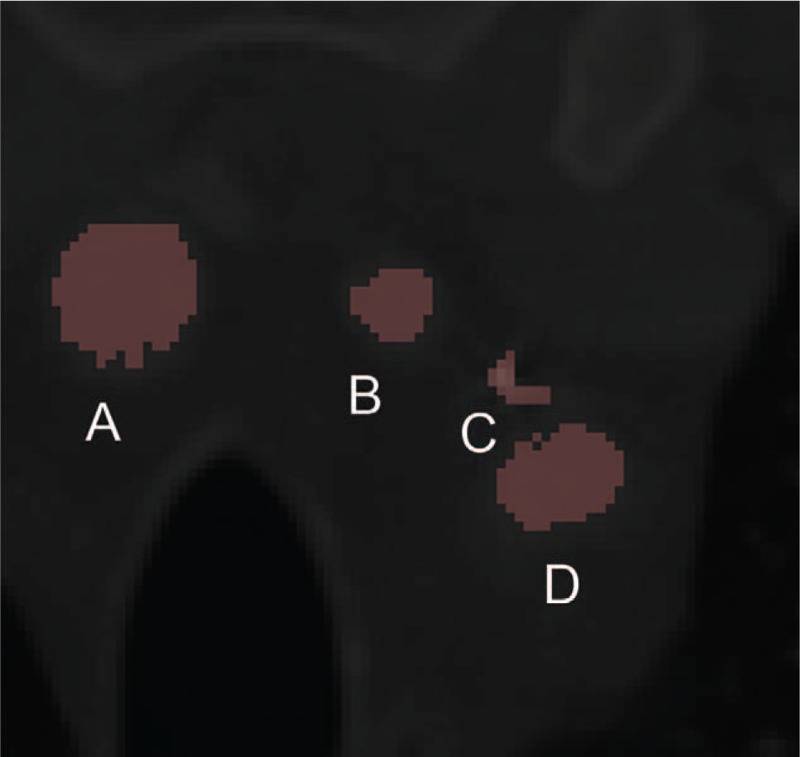
The relative position between brachiocephalic artery (A), left common carotid (B), guide wire (C), and left subclavian artery (D).

At this point, the patient presented with swollen ankle and headaches, and as a result, he wished to have the guidewire removed. The patient then underwent a comprehensive CT scan, which revealed that the guide wire had broken into 2 segments. One segment (with a diameter and length of 2.0 mm and 535 mm, respectively) had arrived at the right brachiocephalic vena cava, and the other segment (with a diameter and length of 1.2 mm and 323 mm, respectively) had arrived at the cerebrum (Figs. 1 and 2). Both the diameter and the length are measured by CT (General Electric Company) and its post-processing platform. The CT scan demonstrated that the core wire had punctured the vascular wall of the aortic arch between the left common carotid artery and the left subclavian artery to reach the cerebrum (Figs. 3–5). There was no obvious thrombus on the ultrasonography. The vascular surgeons, neurosurgeons, and radiologists determined that the risks of surgery to remove the guide wire were high; the patient refused surgery due to worry about the risks outline to him. He was advised to take anticoagulants, avoid strenuous exercise, return for a follow-up visit once every 3 months, and visit the emergency department if his headaches became more painful. The follow-up has for last 5 months by phone message, but the patient did not come to the outpatient department again. The patient has survived for almost 4 years with the guide wire present in his circulation.

## 3 Discussion

Central venous access techniques are commonly used for both diagnosis and treatment, especially in the emergency room and the intensive care. However, central venous catheterization has many serious complications such as infection, failure to place the catheter, arterial puncture, improper catheter position, misplacement, kinking, breakage, thrombosis, embolism, arrhythmias, pneumothorax, and hematoma, which may arise in as many as 15% of these procedures.^[1,4,5]^ Our study reports a case of a retained guide wire which had broken into 2 segments. In the described case, the wires were retained in the right iliac vein at the point of bifurcation and in the cerebrum. From the lengths and diameters, we estimate that 1 of the 2 wires was the core wire of the guide wire and another was the outer wire. The diameter of the wire in the cerebrum was smaller than that in the right femoral vein. Therefore, we infer that the wire in the cerebrum was the core wire. An important point in preventing this rare complication is that the guide wire should be held at the tip at all times and if the guide wire is lost in the vein, it should be removed as soon as possible.^[2,6]^ If this rule is followed, the guide wires cannot get lost or stay in the vein for such a length of time that makes their removal dangerous.

This is a rare and completely avoidable complication of central venous catheterization. The guide wire should be held at the tip at all times to prevent passage into the vessel.^[6,7]^ Safety measurements with a standard protocol including time out and sign out checklists as mentioned by the World Health Organization, and directly supervised bedside teaching are mandatory measures in order to prevent the occurrence of such complications.^[8]^ Bedside teaching is defined as a minimum of 2 clinicians (resident and supervisor) who can remind each other about removal of the guide wire communicate about the different steps during the central venous catheterization, and check the CT scans. Finally, counting the equipment after the procedure as also stated in the World Health Organization sign out guideline is of crucial importance to prevent errors. During central venous catheterization, guide wire-related complications are uncommon and essentially preventable. The following precautions should be taken:^[8]^

- Inspect the wire for defects before insertion
- Consider a guide wire to be a delicate and fragile instrument
- When resistance to insertion is met, remove and inspect the wire for damage, reposition the introducer so that no resistance to its passage is felt
- Particular caution should be used when attempting central catheter placement in patients who are predisposed to thrombosis or have had repeated catheterizations of a particular vessel
- If multiple manipulations are required, reinspect the wire and replace it if necessary
- Pass the catheter over the wire into the vein
- Ensure that the wire is visible at the proximal end before the catheter is advanced
- The catheter should be “railroaded” over the guide wire into the vein, holding the wire, and not pushing catheter and wire together into the vein
- Always inspect the wire for complete removal at the end of the procedure
- Hold onto the wire at all times until it is removed from the vessel.

In 2002, Schummer W reported 4 cases that showed predisposing factors for incorrect intravascular placement of a guide wire; these included inattention, inexperienced—operators, either in terms of their methods or in the central venous cannulation, inadequate supervision of trainees and overtired staff.^[8]^ In the current case, pit is a likely that a combination of these factors led to the guide wire going unnoticed and left in the patient.

Removal of the foreign body is critical even if the patient is asymptomatic because of possible life-threatening complications both in the acute setting and in the long term.^[9,10]^ If the wire has remained in the vessel for several years, removing the foreign body is likely to damage the vessel due to possible neo-intimalization of the wire.^[11]^ Retained guide wires can be removed by interventional radiology with the help of endovascular forceps and loop snare.^[12,13,14]^ The loop snare technique is the method of choice in most of the cases because of its safety, feasibility, relative ease of use, high success rate with low complication rates, and its cost-effectiveness.^[13,14]^ Unless the patient's condition doesn’t allow percutaneous treatment to be performed, percutaneous treatment is more safe and effective as compared to open vascular surgery.^[6,9,12,15]^

The perforation of central veins or cardiac chambers can be catastrophic. In clinical practice, the venous perforation is either caused by the introducer needle, guide wire, or the dilator. Previous literature reports cases of guide wire related perforation of the great vessels, including the brachiocephalic and subclavian veins.^[16]^ This case of perforation of arch of aorta and cerebrum caused by the core wire has never been previously reported. This important complication occurs when the resistant force of the core wire, caused by hemokinesis, is applied against the arch of aorta. In most instances, bleeding from a small penetrating hole in a vein will stop spontaneously either by vasospasm or by external compression of the surrounding tissues.^[16]^ This theory explains the reason why the described patient remains alive, albeit with persistent headaches. Alternatively, we suggest another hypothesis that the neo-intimalization of the wire due to the additional coverage of endothelial cells in the aortic arch blocked the perforation caused by the wire. With regards to how the core wire gets to the artery from the vein, we suggest that due to the resistant force caused by hemokinesis, the core wire went through the thinner atrial septum; unfortunately, we could not find any evidence to prove this hypothesis. Regarding the swelling of the right ankle joint, we do not consider that this was caused by the guide wire, since we found that the deep vein of the right lower extremity was unobstructed. Because the removal of the guide wire is problematic and may lead to the eventual death of the patient, the attendance at follow-up visits is crucial.

## 4 Conclusion

Although central venous puncture is a frequently performed procedure especially in interventional therapy, we wish to emphasize and raise awareness of potential complications. The loss of a guide wire is a completely preventable complication, provided that one maintains a hold of the tip of the wire during placement, and that correct safety measurements and protocols are followed. The vasospasm, neo-intimalization and the external compression of the surrounding tissues play an important role in stopping the bleeding from the perforation site caused by the wire.

## Author contributions

**Conceptualization:** Yu Zhao.

**Data curation:** Shenyu Zhao.

**Formal analysis:** Yu Zhao.

**Project administration:** Shenyu Zhao, Zhe Wang.

**Resources:** Shenyu Zhao, Zhe Wang, Yu Zhao.

**Supervision:** Yu Zhao.

**Visualization:** Zhe Wang.

**Writing – original draft:** Shenyu Zhao.

**Writing – review & editing:** Shenyu Zhao, Zhe Wang.
